# Cisplatin Neurotoxicity Targets Specific Subpopulations and K^+^ Channels in Tyrosine-Hydroxylase Positive Dorsal Root Ganglia Neurons

**DOI:** 10.3389/fncel.2022.853035

**Published:** 2022-05-02

**Authors:** Carrie J. Finno, Yingying Chen, Seojin Park, Jeong Han Lee, Maria Cristina Perez-Flores, Jinsil Choi, Ebenezer N. Yamoah

**Affiliations:** ^1^Department of Population Health and Reproduction, School of Veterinary Medicine, University of California, Davis, Davis, CA, United States; ^2^Department of Physiology and Cell Biology, School of Medicine, University of Reno, Reno, NV, United States

**Keywords:** cancer, chemotherapy, neuropathy, sensory, vitamin E

## Abstract

Among the features of cisplatin chemotherapy-induced peripheral neuropathy are chronic pain and innocuous mechanical hypersensitivity. The complete etiology of the latter remains unknown. Here, we show that cisplatin targets a heterogeneous population of tyrosine hydroxylase-positive (TH^+^) primary afferent dorsal root ganglion neurons (DRGNs) in mice, determined using single-cell transcriptome and electrophysiological analyses. TH^+^ DRGNs regulate innocuous mechanical sensation through C-low threshold mechanoreceptors. A differential assessment of wild-type and vitamin E deficient TH^+^ DRGNs revealed heterogeneity and specific functional phenotypes. The TH^+^ DRGNs comprise; fast-adapting eliciting one action potential (AP; 1-AP), moderately-adapting (≥2-APs), in responses to square-pulse current injection, and spontaneously active (SA). Cisplatin increased the input resistance and AP frequency but reduced the temporal coding feature of 1-AP and ≥2-APs neurons. By contrast, cisplatin has no measurable effect on the SA neurons. Vitamin E reduced the cisplatin-mediated increased excitability but did not improve the TH^+^ neuron temporal coding properties. Cisplatin mediates its effect by targeting outward K^+^ current, likely carried through K2P18.1 *(Kcnk18*), discovered through the differential transcriptome studies and heterologous expression. Studies show a potential new cellular target for chemotherapy-induced peripheral neuropathy and implicate the possible neuroprotective effects of vitamin E in cisplatin chemotherapy.

## Introduction

Cisplatin is one of the most efficacious chemotherapeutic drugs to treat several solid and blood cancers (Tsang et al., 2009). Still, it is wrought with peripheral neuropathic side effects that limit its chemotherapeutic value. The anti-cancer effects of cisplatin and related platinum compounds stem from the induction of intra- and inter-strand DNA crosslinks and denaturation of nuclear and mitochondrial DNA. The ensuing effects are necrotic and apoptotic cell death of cancer cells (Eastman and Barry, 1987; Dzagnidze et al., 2007). Puzzlingly, the underlying mechanism for cisplatin-mediated neurotoxicity remains unclear. Symptoms include painful paresthesia in the extremities and thermal and tactile allodynia or hyperalgesia (Hu S. et al., 2019), with dorsal root ganglion neurons (DRGNs) serving as the unquestionable target. The diversity of neuropathic repercussions may stem from DRGNs’ heterogeneity in properties and functions and varying susceptibility to cisplatin toxicity. Studies have shown that cisplatin-mediated degeneration of large-myelinated DRGNs underlies patients’ peripheral neuropathy (Cavaletti et al., 1992; Krarup-Hansen et al., 1999, 2007; McDonald et al., 2005), but the etiology for exaggerated mechanosensitivity is unknown (Starobova and Vetter, 2017). The knowledge gap was one of the motivations for the current studies.

DRGNs are heterogeneous in size, with distinct neurochemistry, and can be grouped into as many as thirteen clusters based on their gene expression, subserving multiple sensory modalities (Usoskin et al., 2015; Li et al., 2016; Renthal et al., 2020). Among the classes of DRGNs are tyrosine-hydroxylase positive (TH^+^) primary afferents (Brumovsky et al., 2006). In adult murine DRGNs, the expression of TH is a defining feature of unmyelinated C-low threshold mechanoreceptors (C-LTMRs) (Li et al., 2011; Kupari and Airaksinen, 2014). An additional marker of this C-LTMR population in adult mice is vesicular glutamate 3 (vGluT3). Although vGluT3 is not a consistent marker of TH^+^ DRGNs during development (Sharma et al., 2020), once adulthood is reached, more than 80% of TH^+^ DRGNs in adult mice express vGluT3 mRNA (Li et al., 2011). Additionally, direct recording from vGluT3^+^ neurons in adult sensory ganglia identified these neurons as C-LTMRs (Seal et al., 2009). Of note, while C-LTMRs label as TH^+^ in mice, they do not in humans (Brumovsky, 2016). However, recent comparative transcriptomic studies demonstrate that TH^+^ DRGNs in mice correspond to C-LTMRs in non-human primates (Kupari et al., 2021) and humans (Tavares-Ferreira et al., 2021).

Across species, C-LTMRs are perceived to respond to the affective component of touch and injury-induced mechanical hypersensitivity (Seal et al., 2009; Olausson et al., 2010). However, in models of acute mechanical pain, chronic inflammatory pain and chemotherapy-induced peripheral neuropathy (CIPN), a change in sensation conveyed by C-LTMRs occurs, from pleasant touch to pain (Seal et al., 2009). These dual functions of C-LTMRs may be mediated, in part, by a chemokine-like secreted protein TAFA4 (*Fam19a4*), which is specifically expressed in C-LTMRs (Delfini et al., 2013). Using *VGluT3*^+^-*channelrhodopsin 2* mice, Draxler et al. (2014) identified three populations of VGluT3^+^ primary afferents, A-fibers, TH^+^ C-LTMRs and TH- C-fibers, that contributed to the development of mechano-cold hypersensitivity in a CIPN model using oxaliplatin. Subsequently, putative c-LTMRs were identified as the subset of DRGNs with the highest number of differentially expressed genes in an animal model of paclitaxel-induced allodynia (Renthal et al., 2020), another chemotherapeutic agent that causes peripheral neuropathy (Toma et al., 2017). Thus, C-LTMRs may be uniquely affected in CIPN.

Previous reports have suggested functional heterogeneity within murine TH^+^ DRGNs (Delfini et al., 2013; Renthal et al., 2020), and unsupervised classification of DRGNs in mice has identified two molecular subsets of TH^+^ neurons; TH1 and TH2. The TH2 neurons were most altered with vitamin E deficiency (Finno et al., 2019). These subsets correspond to a subsequent report by Renthal et al. (2020) that defined a *Th^+^/Fam19a4^+^* C-LTMR (cLTMR1) population and putative second *Fam19a4*^+^ C-LTMR (p_C-LTMR2) population, that was lower in *Th* expression, in wild-type mice. This p_cLTMR2 subpopulation had the most differentially expressed genes across all DRGN populations in mice administered paclitaxel (Renthal et al., 2020). Thus, two subpopulations of C-LTMRs may exist in the adult mouse sensory ganglia that demonstrate differential responses to both chemotherapeutics and vitamin E.

The importance of vitamin E in TH^+^ DRGNs in CIPN is emerging, underpinned by the findings that decreased plasma vitamin E levels increased susceptibility to peripheral neuropathy (Bove et al., 2001). The stark resemblance between the clinical and neuropathologic features of CIPN (Muller et al., 1983; Traber et al., 1987) and patients with peripheral neuropathy due to familial ataxia with vitamin E deficiency (AVED) (Gotoda et al., 1995; Yokota et al., 1996) raises the possibility that the two sensory deficits share a common underlying mechanism.

We tested the hypothesis that the heterogeneity in molecular features of TH^+^ DRGNs yields functional diversity, and the shared characteristics of CIPN and vitamin E deficiency can be used to identify therapeutic targets for sensory deficits. We show that TH^+^ DRGNs consist of at least three functionally distinct neuronal subtypes, two adapting and one spontaneously active (SA) neuronal class, with differential responses to the effects of cisplatin and vitamin E. We demonstrated that the neuroprotective effects of vitamin E on CIPN might be mediated through effects on a two-pore domain subfamily channel, K_2P_18.1 *(Kcnk18*), thus providing a potential therapeutic target for the treatment of sensory deficits associated with CIPN and vitamin E deficiency.

## Materials and Methods

### TH1 vs. TH2 Dorsal Root Ganglion Neuron Single-Cell RNA Sequencing Analysis

To define the differences between TH1 and TH2 DRG neuronal subpopulations, we performed targeted analyses on our previously published single-cell RNA sequencing dataset (Finno et al., 2019). Briefly, this study had performed single-cell RNA sequencing on DRG cells from wild-type C57BL6/J mice and tocopherol transfer-alpha protein null mice (*Ttpa*^–/^*^–^*) to investigate the effect of vitamin E deficiency on DRGN gene expression. Mice were 5–6 months of age and consisted of 1 male and 1 female mouse from three experimental groups; *Ttpa*^+/+^ fed a regular diet (WT), *Ttpa*^–/^*^–^* mice fed a vitamin E deficient diet (DEF), and *Ttpa*^–/^*^–^* mice fed a highly vitamin E supplemented diet (SUPP). In this previous study, unsupervised single-cell transcriptome profiling identified 14 initial subpopulations in all mice (Finno et al., 2019). These clusters were classified based on previous classifications (Usoskin et al., 2015) as peptidergic (PEP1 and PEP2), non-peptidergic (NP1, NP2, NP2-2, and NP3), neurofilament (NF1, NF2, NF3, and NF4-5), tyrosine hydroxylase positive (TH1 and TH2) and “Unassigned” for the unassigned cluster (Finno et al., 2019).

For the current study, we first evaluated two specific markers of C-LTMRs in adult mice, *Slc17a8* (VGluT3) (Seal et al., 2009; Li et al., 2011) and *Fam19a4* (TAFA4), in our dataset. Next, two analyses were performed to further investigate the distinct profiles of TH1 vs. TH2 DRGNs in this dataset. The first analysis determined the number of genes unique to a single cluster when TH1 and TH2 subtypes were compared against all other DRGN subpopulations. This analysis was performed in Seurat using the FindAllMarkers option (Satija et al., 2018). The second analysis compared only TH1 vs. TH2 transcriptional profiles, using a logfc. threshold of 0.25. For both analyses, subsequent pathway analyses were performed using Panther Pathway overrepresentation analysis.^1^ Lastly, we evaluated this dataset for K^+^ channels that were significantly upregulated with vitamin E deficiency in TH^+^ DRGNs, but not previously explored, including the K^+^ two-pore (K_2P_) domain subfamily channels (*Kcnk3, Kcnk12*, and *Kcnk18*).

### Single-Molecule Fluorescence *in situ* Hybridization With RNAscope

Dorsal root ganglion neurons were isolated from TH-EGFP-positive (TH-EGFP+) transgenic mice (Matsushita et al., 2002), using a combination of enzymatic and mechanical procedures. Adult male and female (6–8 weeks old) TH-EGFP+ mice were euthanized and DRG neurons collected. Dorsal root ganglion neurons were dissected and fixed in 4% PFA in DEPC-treated PBS for 2 h at 4°C to preserve RNA. Samples were washed with DEPC-treated PBS three times. DRG samples were sequentially dehydrated in 10, 20, and 30% sucrose solution at 4°C for 1 h, 2 h, and overnight, respectively, then embedded in OCT for cryosection. Samples were cryo-sectioned to a thickness of 10 μm, placed onto Superfrost slides, and stored at −80°C until processed. Probe hybridization was performed according to the manufacturer’s instructions (Advanced Cell Diagnostics, ACD). Sections were immersed in pre-chilled 4% PFA for 15 min at 4°C. Sections were then dehydrated at RT in 50%, 70%, and twice in 100% ethanol for 5 min each and allowed to dry for 1–2 min. Fixation and dehydration were followed by protease digestion, using protease for 30 min at RT. Sections were then incubated at 40°C with the following solutions: (1) target probe in hybridization buffer A for 3 h; (2) preamplifier in hybridization buffer B for 30 min; (3) amplifier in hybridization buffer B at 40°C for 15 min; and 4) label probe in hybridization buffer C for 15 min. After each hybridization step, slides were washed with washing buffer three times at RT. For fluorescent detection, the label probe was conjugated to Alexa Fluor 594. Probes, positive and blank negative controls were obtained from ACD. Sequences of the target probes, preamplifier, amplifier, and label probe are proprietary. Detailed information about the probe sequences can be obtained by signing a non-disclosure agreement provided by the manufacturer. Incubation in DAPI solution for 15 s at RT was performed to label cell nuclei. Slides were then mounted in Fluoromount-G and sealed under a coverslip. Images were captured with a Nikon A1 and Olympus FV1000 confocal microscope. Dots in each fluorescent positive cell were counted and scored as described.

### Cell Culture

Dorsal root ganglia neurons were isolated from TH-EGFP+ mice, as previously described, and the DRG were removed in a solution containing Minimum Essential Medium with HBSS (Invitrogen), 0.2 g/L kynurenic acid, 10 mM MgCl_2_, 2% fetal bovine serum (FBS; v/v), and 6 g/L glucose. The central DRG tissue was dissected and digested in an enzyme mixture containing 1 mg/ml collagenase type I and 1 mg/ml DNase at 37°C for 15 min. After a series of gentle triturations and centrifugation in 0.45 M sucrose, the cell pellets were reconstituted in 900 μl of culture medium (Neurobasal-A, supplemented with 2% B27 (v/v), 0.5 mM L-glutamine, and 100 U/ml penicillin; Invitrogen), and filtered through a 40 μm cell strainer for cell culture and electrophysiological experiments. For adequate voltage-clamp and satisfactory electrophysiological experiments, we cultured DRGNs for ∼24 h. All electrophysiological experiments were performed at room temperature (RT; 21–22°C). To validate the TH-EGFP+ model in DRGNs, we performed smFISH with RNA-scope, as described previously, using RNA-scope probes for GFP (ACD, Cat. No. 409011) and TH (ACD, Cat. No. 317621) overlayed with Tuj1 antibody for neurons (Biolegend, Cat. No. 802001).

### Electrophysiology

Whole-cell membrane potential recordings were performed using an Axopatch 200B amplifier (Molecular Devices, Sunnyvale, CA, United States). Membrane potentials were amplified, bandpass filtered (2–10 kHz), and digitized at 5–50 kHz using an analog-to-digital converter (Digidata 1200, Molecular Devices) as described earlier (Levic et al., 2007; Rodriguez-Contreras et al., 2008). Electrodes (2–3 MΩ) were pulled from borosilicate glass pipettes, and the tips were fire-polished. The normal extracellular/bath solution consisted of (in mM) 130 NaCl, 5 KCl, 1 MgCl_2_, 2 CaCl_2_, 10 D-glucose, and 10 4-(2-hydroxyethyl)-1-piperazineethanesulfonic acid (HEPES), pH 7.3. The normal internal/pipette/solution contained (in mM) 132 KCl, 1 MgCl_2_, 0.01 CaCl_2_, 2 ethylene glycol-bis(β-aminoethyl ether)-N,N,N′,N′-tetraacetic acid (EGTA) 5 ATP-K_2_, and 10 HEPES, pH 7.3. The seal resistance was typically 5–10 GΩ. Capacitance and series resistance compensation (>90%) were made, and traces were filtered at 2 kHz using an 8-pole Bessel filter and sampled at 5 kHz. The liquid junction potentials (LJP) were measured (2.1 ± 1.2 mV; *n* = 87) and corrected as described previously (Rodriguez-Contreras and Yamoah, 2001). Data analyses were performed using the pClamp and Origin software (MicroCal Inc., Northampton, MA, United States). Where appropriate, pooled data are presented as mean ± SD. To allow for the evaluation of voltage-independent K^+^ currents, leak-current subtraction protocols were not used.

### Statistical Analyses

Where appropriate, pooled data are presented as mean ± SD. Significant differences between groups were tested using *t*-test and ANOVA, where applicable. The null hypothesis was rejected when the two-tailed *p*-value <0.05 is indicated with *, <0.01 with ^**^, <0.001 with ^***^ and <0.0001 with ^***^. The number of mice and neurons are reported as *n*.

### Study Approval

Animals were housed and cared for under the University of California Davis (UCD) and University of Reno (UNR) standing committee on animal use and care (IACUC) as well as the Guide for the Care and Use of Laboratory Animals (8th edition, 2011). All procedures performed were also approved by the University (UCD and UNR) IACUC.

## Results

### Transcriptomic Profiling of Murine TH^+^ Dorsal Root Ganglion Neuron Subpopulations

**FIGURE 1:**
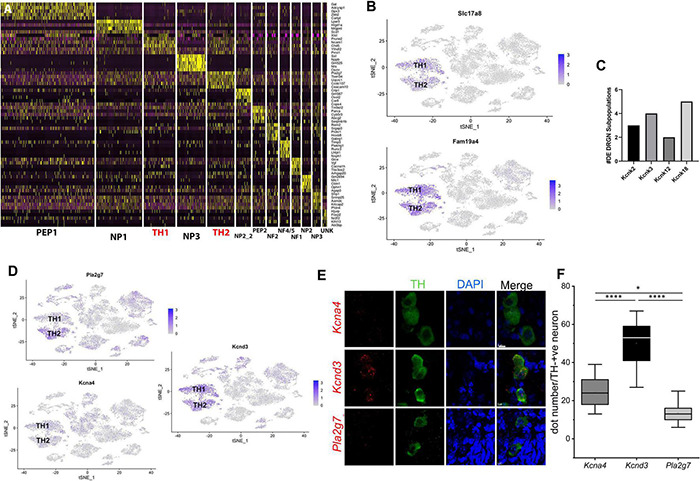
TH1 and TH2 are distinct subpopulations of TH^+^ DRGNs of adult mice. **(A)** Using single-cell RNA-sequencing data from our previously published study (Finno et al., 2019), a heat map of the top five unique genes defining each DRGN subpopulation was generated to clearly define the differences in transcriptional profiles of the two TH^+^ DRGN subpopulations (TH1 and TH2; red). **(B)** TSNE plots demonstrating overall expression of *Slc17a8* (VGluT3) and *Fam19a4* (TAFA4). **(C)** Differential transcript expression of K^+^ two-pore domain subfamily K channels (*Kcnk2, Kcnk3, Kcnk12*, and *Kcnk18*) across the DRGN subpopulations, with *Kcnk18*, differentially expressed across five subpopulations [peptidergic 1 (PEP1), peptidergic 2 (PEP2), TH1, TH2, unknown (UNK)]. **(D)** TSNE plots demonstrating overall expression of *Pla2g*7 (TH2 > TH1), *Kcna4* (TH1 > TH2), and *Kcnd3* (TH1 > TH2) from single-cell RNA-sequencing (Finno et al., 2019). DE, differentially expressed; NF, neurofilament; NP, non-peptidergic; PEP, peptidergic; TH, tyrosine hydroxylase; UN, unknown cluster. *n* = 2 mice per group with ∼3,600 cells/mouse profiled. **(E)** Single-molecule fluorescence *in situ* hybridization (smFISH) with RNAscope from TH-EGFP+ mice, demonstrating expression of *Kcna4*, *Kcnd3*, and *Pla2g7* in TH^+^ DRGNs. Green: TH^+^ DRGN, Blue: DAPI nuclear stain, Red: *Kcna4* (first row), *Kcnd3* (second row), and *Pla2g7* (third row) mRNA. Scale bars represent 5 μm. **(F)** Quantification of mRNA using RNAscope for *Kcna4*, *Kcnd3, and Pla2g7*. Within TH^+^ DRGNs, *Kcnd3* was the most highly expressed. Mean ± SD, *N* = 11 neurons per experimental group (3 mice in each group), one-way ANOVA, or Kruskal-Wallis. Compared to GAPDH (not shown) one-way ANOVA, *p < 0.05, ^****^p < 0.0001.

Our previously published single-cell RNA-sequencing study evaluated the effect of vitamin E on DRGN gene expression and revealed distinct TH^+^ neurons (Finno et al., 2019). Despite highly overlapping transcriptional profiles within the TH^+^ DRGN group, at least two subtypes were not closely related enough to merge, resulting in additional distinction into TH1 and TH2 subgroups. In particular, profound alterations with vitamin E deficiency were identified in the TH2 subpopulation (Finno et al., 2019). Therefore, for the current study, we hypothesized that distinct molecular identity may yield diverse functional phenotypes amongst the TH^+^ DRGNs, that albeit, likely confer varied pharmacology. To illustrate the difference in transcriptional profiles between the TH1 and TH2 subpopulations, the top five transcripts defining each DRGN subpopulation from the previously reported dataset (Finno et al., 2019) are represented in Figure 1A, with TH1 and TH2 subpopulations delineated in red. Next, we determined the relative expression of two markers of C-LTMRs in adult mice, *Slc17a8* (VGluT3) (Seal et al., 2009; Li et al., 2011) and *Fam19a4* (TAFA4). Both markers were highly expressed in both TH1 and TH2 subpopulations (Figure 1B), confirming their presence in adult murine TH^+^ C-LTMRs.

We then used this previously generated single-cell RNA-sequencing dataset (Finno et al., 2019) to determine the number of genes unique to a single cluster when TH1 and TH2 groups were compared against other DRGN subpopulations. Within the TH1 subpopulation, 102 uniquely expressed genes were identified and 254 unique genes were identified in the TH2 subpopulation (Supplementary Table 1). Upregulation of voltage-gated Ca^2+^ and K^+^ channels has been detected with vitamin E deficiency, specifically in the TH2 subpopulation, including Ca_v_2.3 and the Ca^2+^-activated K^+^ channels, Kcnmb1 and Kcnmb2 (Finno et al., 2019). For the current study, we evaluated the remaining K^+^ channels that were significantly upregulated with vitamin E deficiency in TH^+^ DRGNs, including the K^+^ two-pore (K_2P_) domain subfamily channels (*Kcnk3, Kcnk12*, and *Kcnk18*). Of these, *Kcnk18* was differentially expressed across five DRGN subpopulations (Figure 1C), with more significant upregulation in TH2 neurons (*p* = 4.74 × 10^–5^) than TH1 (*p* = 0.01). This channel was thus prioritized for further investigation.

The top TH2 unique transcript, *phospholipase A2 group 7* (*Pla2g7)*, was overrepresented in the TH2 subpopulation when evaluating expression using the TSNE plot (Figure 1D). For the TH1 subpopulation, the top transcripts included peripherin (*Prph*) and ribosomal proteins, which were not very specific when evaluating the TSNE plot (Supplementary Figure 1). Of the differentially expressed K^+^ channels, K_v_1.4 (*Kcna4*) significantly defined the TH1 subpopulation (*p* = 0.0001; Figure 1D). Pathway analysis for the unique genes in the TH1 subpopulation did not identify any statistically significant pathways (P_FDR_ < 0.05). In contrast, a 10.07-fold enrichment of the ubiquitin-proteasome pathway (P_FDR_ = 0.007) was placed in the TH2 subpopulation.

The second analysis compared only the TH1 vs. TH2 transcriptional profiles. A total of 1,052 differentially expressed transcripts were identified, with 310 higher in TH1 and 742 higher in TH2 (Supplementary Table 2). Of the differentially expressed K^+^ channels, K_v_4.3 (*Kcnd3*) was significantly increased in the TH1 vs. TH2 subpopulation (*p* = 6.17 × 10^–21^; Figure 1D). The 310 transcripts that were higher in TH1 DRGNs corresponded to 18 upregulated pathways, including hedgehog signaling, the three metabotropic glutamate receptor pathways, and many G-protein-related receptor signaling pathways (Supplementary Figure 2A). The 742 transcripts that were higher in TH2 DRGNs were associated with two upregulated pathways, Parkinson’s disease (P_FDR_ = 1.02 × 10^–7^) and the ubiquitin-proteasome pathway (P_FDR_ = 0.003) (Supplementary Figure 2B). To validate these top differentially expressed transcripts (*Pla2g7*, *Kcna4*, and *Kcnd3*) in TH^+^ DRGNs, RNA-scope was performed (Figure 1E). All transcripts were expressed in TH^+^ DRGNs, with *Kcnd3* expressed highest (Figure 1F). These results support the distinction of at least two TH+ subpopulations of DRGNs in adult mice and prioritized three K^+^ channels, K_2P_18.1 (*Kcnk18*), K_v_1.4 (*Kcna4*), and K_v_4.3 (*Kcnd3*), for further experiments.

### Electrophysiological Recordings of TH^+^ Dorsal Root Ganglion Neurons

To test the hypothesis that the molecular heterogeneity in TH^+^ DRGNs yield functional diversity, we examined their response properties using TH-EGFP-positive (TH-EGFP+) transgenic mice. Data were obtained from isolated TH-EGFP+ DRGNs from adult (6–8-week-old) male and female mice. To validate this model in DRGNs, we first demonstrated overlap between GFP and TH^+^ C-LTMRs in adult mice (Supplementary Figure 3).

**FIGURE 2:**
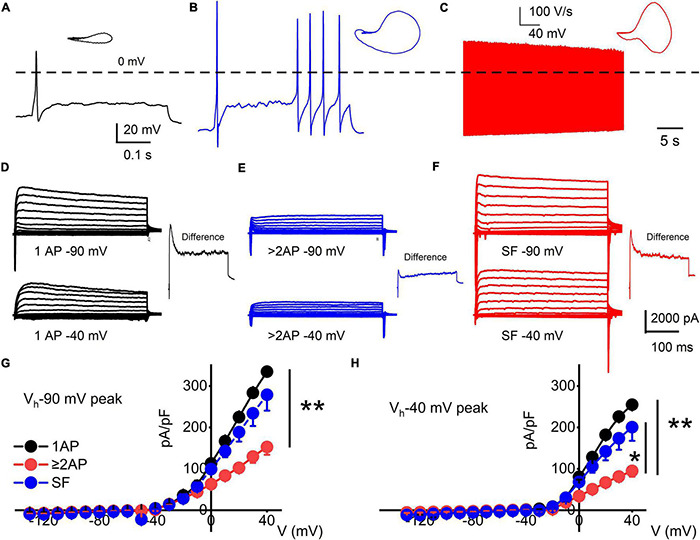
Functional differences in response properties of TH^+^ DRGNs. **(A–C)** Representative AP traces were recorded from TH-EGFP+ transgenic mice DRGNs. **(A)** Fast-adapting TH^+^ DRGNs elicit 1-AP upon current injection (0.2 nA for the example shown). **(B)** Moderately-adapting TH^+^ DRGNs, eliciting ≥2-APs (0.2 nA). **(C)** Spontaneously active (SA) TH^+^ DRGNs. **(D–F)** Whole-cell K^+^ currents in 1-AP **(D)**, ≥2-APs **(E)**, and SF **(F)** TH^+^ DRGNs. Voltage-clamp recordings of TH^+^ DRGNs following current-clamp assessment. TH^+^ DRGNs were held at −90, and −40 mV stepped from −100 to 40 mV using 10-mV increments. To differentiate between ion channels that have different activation potentials, the insets show a difference-current trace using 40-mV stepped potential between neurons held at −90 and −40 mV. **(G,H)** Summary data for the current-voltage (I/V) relationship showing differences in the current densities between the three classes of TH^+^ DRGNs. TH^+^ DRGNs with 1-AP (in black symbols), ≥2-APs (in red symbols), and SF (in blue symbols). (*p < 0.05; **p < 0.01; *n* = 15 neurons from four mice).

Three functional subtypes of TH^+^ DRGNs were identified (Figure 2). The first subtype included fast-adapting TH^+^ DRNGs that elicited one action potential (1-AP) upon current injection (Figure 2A). The second included moderately adapting TH^+^ DRGNs that produced greater than two APs (≥2-APs) upon current injection (Figure 2B). The third subtype was spontaneously active (SA) TH^+^ DRGNs (Figure 2C). The three functionally distinct TH^+^ DRGNs showed stark differences in membrane input resistances and AP properties, sufficient to consider them separate (see Figure 2 legend). To further define the three classes of TH^+^ DRGNs, we switched from current- to voltage-clamp and measured the underlying whole-cell K^+^ current in the TH^+^ DRGN subtypes (Figures 2D–F). Held at −90 and −40 mV and stepped from −100 to 40 mV using 10-mV increments, the yielding outward currents varied across the TH^+^ DRGNs (Figures 2G,H). The two holding potentials were used to conduct a coarse assessment of the inactivating and non-inactivating K^+^ currents. Inward current evaluation was not feasible under the experimental configurations.

**FIGURE 3:**
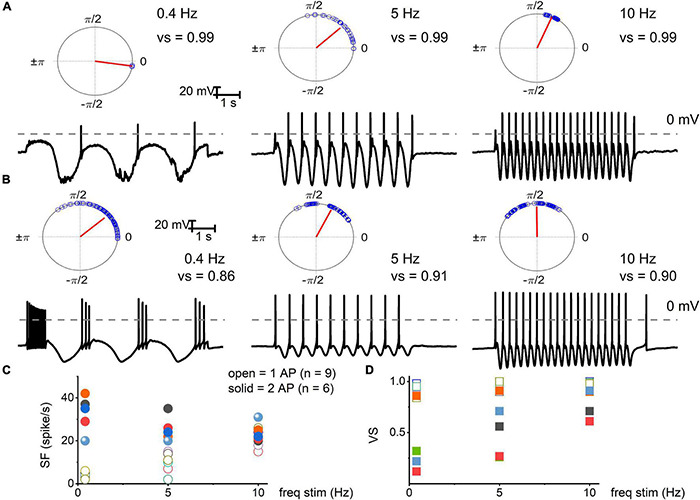
Electrical stimulation of TH^+^ DRGNs with oscillatory current injections. **(A)** Upper panel: polar plots for TH^+^ DRGNs with a 1-AP-elicited square pulse. Below are representative membrane responses of TH^+^ DRGNs to oscillatory current injection (0.2 nA) at 0.4, 5, and 10 Hz. **(B)** Row of similar polar plots derived from TH^+^ DRGNs responding with ≥2-APs after square pulse injection (0.2 nA) to 0.4, 5, and 10 Hz oscillatory currents. **(C,D)** Summary data of the two sets of TH^+^ DRGNs, showing the relationship between oscillatory current injection (0.4, 5, and 10 Hz, 0.2 nA) and spike frequency (open and solid symbols represent data from neurons with 1 AP, and ≥2 AP response features, respectively). **(D)** Data were obtained from the same neurons as in **(C)**, with computed vector strength (VS). 1-AP TH^+^ DRGNs consistently showed higher VS, indicating better temporal coding. Each symbol represents a different TH^+^ DRGNs (*n* = 9 for 1-AP, *n* = 6 for ≥2-APs TH^+^ DRGNs).

We used oscillatory current injections at 0.4, 5, and 10 Hz as a proxy for the frequency regime of natural stimuli to evaluate the response properties of identified TH^+^ DRGNs (Figures 3A,B). In response to sinusoidal current, the spike frequencies were enhanced as stimulus frequency increased in the fast-adapting 1-AP-neurons, reaching a saturating response at >10 Hz (Figure 3C). For moderately-adapting ≥2-APs-neurons, the spike frequency declined, attaining an asymptotic level at >20 Hz (Figure 3C). The vector strength (VS) measures the degree of synchronization between the stimulus and response. A value of 1 refers to perfect phase synchrony, and 0 refers to a random relation between stimulus and response (Goldberg and Brown, 1969). The computed VS for the 1-AP- was consistently greater than ≥2-APs-neurons (cf., a caveat in analyses, Figure 3 legend). Vector strength reduced as the stimulation frequency increased above 20 Hz for the two classes of neurons. However, invariably, the 1-AP-neurons had higher VS, indicating enhanced temporal coding (Figure 3D). Vector strength could not be determined for SF TH^+^ DRGNs due to the continual firing of these neurons. Results support distinct functional roles for three subtypes of TH^+^ DRGNs; 1-AP, ≥2-APs, and SA.

### Cisplatin and Vitamin E-Mediated Alterations of TH^+^ Dorsal Root Ganglion Neurons and Potential K^+^ Channel Target

**FIGURE 4:**
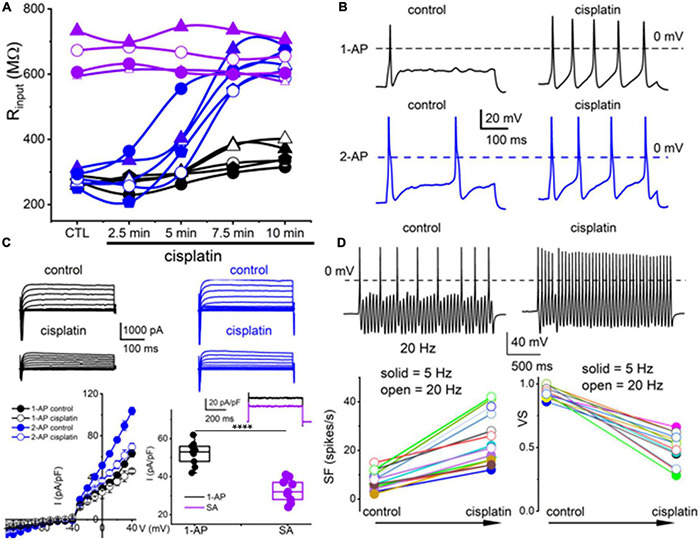
Cisplatin differentially increased membrane excitability of TH^+^ DRGNs but reduced their temporal coding properties. **(A)** Time-dependent alterations of the input resistance of TH^+^ DRGNs after application of 2 μM cisplatin. Black and blue symbols represent TH^+^ DRGNs that elicit 1-AP (*n* = 5) and ≥2-APs (*n* = 5) upon current injection. In purple are spontaneously active neurons (*n* = 4). **(B)** Exemplary effects of cisplatin on rectangular-shaped current-injected APs on two distinct TH^+^ DRGNs [1-AP (upper, shown in black traces, and ≥2-APs (lower, shown in blue traces, panels)]. **(C)** Whole-cell currents from 1-AP (black) and ≥2-APs TH^+^ DRGNs (blue) show cisplatin-mediated outward currents reduction. The left lower panel shows the corresponding current density (pA/pF) and voltage relation (*n* = 9). For outward currents elicited from −70 to 0 mV, cisplatin reduced the current by ∼23% for 1-AP TH^+^ DRGNs and ∼30% for ≥2-APs TH^+^ DRGNs. The middle-lower panel shows the difference in current, or the cisplatin-sensitive current, which appears to exhibit time-independent properties. The inset shows current from 1-AP TH^+^ DRGNs (black), and spontaneously active TH^+^ DRGNs (purple). Summary data from currents generated from a holding potential of −70 mV to step potential of 40 mV. Mean for 1-AP = 52.1 ± 6.23 and spontaneously active neuron = 32.8 ± 6.09 (****p = 5.6 × 10^–6^; *n* = 9 neurons from three mice). **(D)** Representative response properties of 1-AP TH^+^ DRGNs generated by simultaneous sinusoidal current injection (0.2 nA, 20 Hz). The amplitude criterion of a valid AP was 0 mV overshoot (dashed line). The lower panels summarize spike frequencies and vector strength (VS) changes before and after cisplatin (1 mM) application. Data were collected using 5 Hz (*n* = 8) and 20 Hz (*n* = 7) sinusoidal current (***p < 0.001).

The TH^+^ DRGNs are mechanically sensitive and are subject to profound transcriptional modifications in response to vitamin E deficiency (Finno et al., 2019). We surmised that cisplatin might target TH^+^ DRGNs stemming from the drug’s tactile hyperalgesia side effects (Hu S. et al., 2019). As exemplified in Figure 4A, cisplatin had minimal impact on the membrane input resistance of SA TH^+^ DRGNs. By contrast, cisplatin-induced a time-dependent membrane input resistance increase in the 1-AP- and ≥2-AP neurons (Figure 4A). The effects of cisplatin can be seen in the expected increase in spike frequency (SF) and membrane excitability in TH^+^ DRGNs, except for the SA neurons (Figure 4B). Whole-cell currents from 1-AP and ≥2-APs TH^+^ DRGNs showed cisplatin-mediated outward current reduction (Figure 4C). For outward currents elicited from −70 to 0 mV, cisplatin reduced the current by ∼13% for 1-AP TH^+^ DRGNs and ∼30% for ≥2-APs TH^+^ DRGNs (Figure 4C). When a sinusoidal current was injected into a 1-AP TH^+^ DRGN, SF increased (*p* < 0.001), and the VS decreased (*p* < 0.001) after the application of cisplatin (Figure 4D). The differential effects of cisplatin on the TH^+^ DRGN subtypes substantiated the assertion that the neurons may be functionally and pharmacologically different despite a common distinct transcriptional biomarker. Additionally, the significantly large time-independent K^+^ current and cisplatin-sensitive current in 1-AP TH^+^ DRGNs provides insight into the underlying mechanism, which can be attributed to the roles of K2P channels.

**FIGURE 5:**
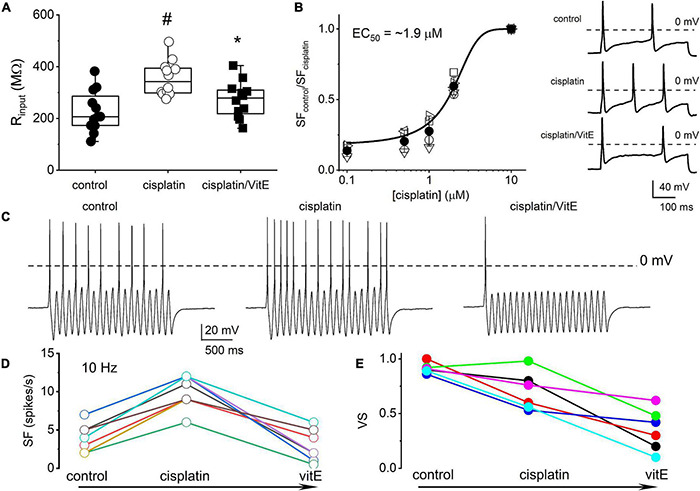
Vitamin E reduces cisplatin-mediated increased excitability of TH^+^ DRGNs but does not improve the coding properties. **(A)** Summary of the effects of cisplatin on the input resistance of TH^+^ DRGNs after application of 1.5 μM cisplatin and the subsequent effects of the application of vitamin E (vitE, 100 μM) and 1.5 μM cisplatin. Differences between control and the treatment were tested using one-way ANOVA. ^#^*p* = 3.75 × 10^–4^ and *p = 2.32 × 10^–2^ (*n* = 12). **(B)** Dose-response relation on cisplatin-mediated increased spike frequency and applied cisplatin concentrations. A Hill coefficient of 2 and EC50 of 1.9 ± 0.3 μM (*n* = 4) was estimated. The inset on the right shows the effects of 0.5 μM cisplatin. **(C)** Effects of cisplatin (0.5 μM) and vitE (100 μM) on the response properties of TH^+^ DRGNs generated by injecting sinusoidal current (0.2 nA, 10 Hz). **(D)** The relation between spike-frequency in control, cisplatin and after cisplatin/vitE application (*n* = 6). Data were assessed from TH^+^ DRGNs that generated 1-AP in response to a square pulse. **(E)** The corresponding computed VS, cisplatin, and vitamin E reduce the VS (*n* = 6).

Because vitamin E has specific marked effects on TH^+^ DRGNs (Finno et al., 2019), we examined vitamin E-mediated alterations of cisplatin-induced excitability, focusing on 1-AP neurons. The addition of cisplatin significantly increased the input resistance of 1-AP TH^+^ DRGNs (*p* = 3.75 × 10^–4^), and the subsequent application of vitamin E reduced the input resistance to baseline levels (*p* = 2.32 × 10^–2^, Figure 5A). The application of vitamin E reduced the cisplatin-induced increased excitability of 1-AP TH^+^ DRGNs (Figures 5B–D) but did not improve the VS (Figure 5E). Results suggest that vitamin E reduces the cisplatin-mediated increase in TH^+^ DRGN excitability but does not improve the temporal coding properties.

**FIGURE 6:**
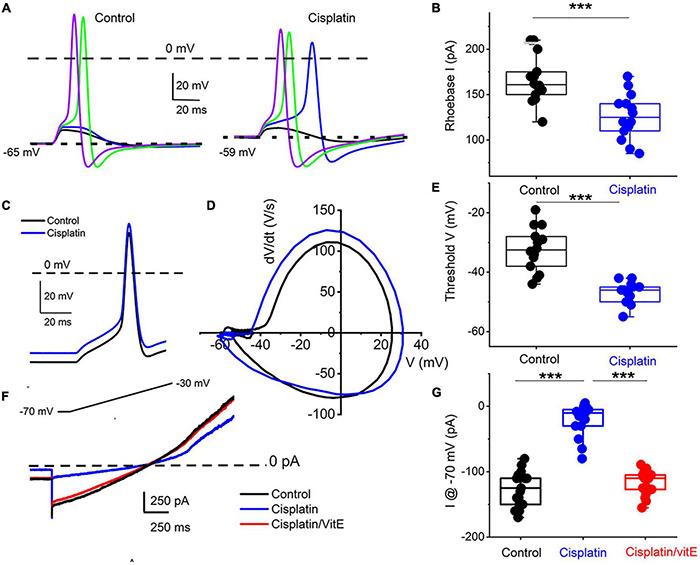
Cisplatin reduced induced membrane potential depolarization and reduced action potential threshold by suppressing a resting current. **(A)** Left panel. Using a 5-ms current injection of different amplitudes, membrane depolarizations and action potentials were generated. The strategy was used to determine the action potential threshold before and after applying 1 μM cisplatin (right panel). The dashed line indicates the 0-mV level, and the dotted line represents the resting membrane potentials. Note that cisplatin mediated ∼5–8-mV membrane depolarization. **(B)** Average and raw data of the effective rheobase evaluated from **(A)** plotted for control and after application of cisplatin (1 μM). *p*-values for statistical comparison are shown, and statistical significance is indicated with an asterisk (***p < 0.001, *n* = 14 neurons from three mice). **(C)** Exemplary action potentials were generated using 150 pA current injection for control (in black) and after cisplatin application (in blue). **(D)** Phase plot (dV/dt) of the action potentials in **C** comparing controls (in black) and cisplatin effect (in blue). The membrane voltage threshold is determined from the phase plots for control and after cisplatin application. Cisplatin reduced the threshold voltage significantly (***p < 0.001) as summarized in **(E)** (*n* = 13 from three mice). **(F)** Current traces showing generated with a voltage-ramp from −70 mV holding voltage to −30 mV for control (black) and in the presence of cisplatin (1 μM, blue) followed by application of solution containing 1 μM cisplatin and 100 μM vitE (red). Cisplatin reduced the holding current, which was reversed with vitE application. Summary of the measured holding current at −70 mV holding voltage in control, after cisplatin and vitE (***p < 0.001, *n* = 16 from four mice).

To determine the properties of the underlying current responsible for cisplatin-mediated effects on 1-AP TH^+^ DRGNs, we examined the resting membrane potential in control and following cisplatin application. For 29 DRGNs studied, cisplatin produced a 6 ± 2 mV depolarizing shift in the resting membrane potential (Figures 6A,B). The rheobase current was reduced as summarized in Figure 6B, suggesting that the underlying current may be partially operating at rest. Analyses of the threshold of all-or-none AP in control and, upon application of cisplatin, revealed a reduced activation threshold in cisplatin (Figures 6C–E). We predicted that the cisplatin-mediated effect likely is active at rest. We switched to voltage-clamped mode and evaluated the effects of cisplatin on the holding current at −70 mV holding potential. Consistent with the expectation, cisplatin reduced the holding current, and applying vitamin E reduced the cisplatin effects on the holding current and the ramp-voltage protocol-induced current, substantiating the prediction that a baseline “leak” current may be blocked by cisplatin (Figures 6F–G).

**FIGURE 7:**
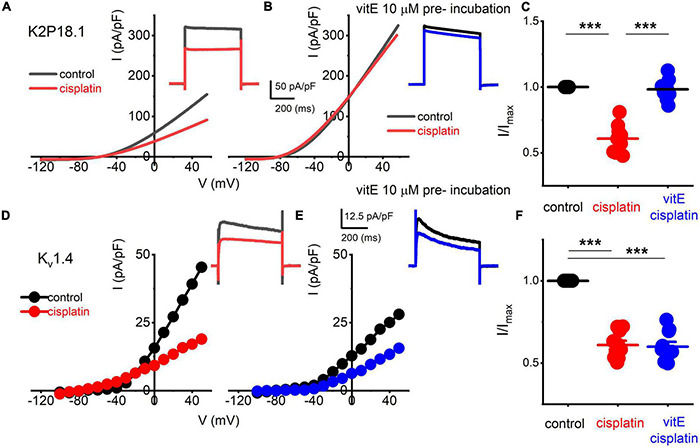
Effects of vitE pre-incubation on cisplatin-mediated alterations on expressed K_2P_18.1 and K_v_1.4 in HEK 293 cells. Outward K^+^ currents from HEK 293 cells transfected with the mouse K_2P_18.1 and K_v_1.4 plasmids. **(A)** K_2P_18.1 channel expression; the recordings were obtained from a holding potential of −80 mV and stepped to −100 mV with a ramp to 60 mV within 500 ms (in black) or a square pulse ranging from −70 to 60 mV (ΔV = 10 mV). The insets show square-pulse elicited current. The red- square and ramp traces depict the effects of cisplatin application (10 μm). Cisplatin (10 μM) blocked ∼40% of the current. **(B)** In contrast, pre-incubation (∼2 h) in 10-μM vitE sufficed to abolish the effects of cisplatin on K_2P_18.1 current (blue). I-V relationship in response to a ramp stimulus. **(C)** Summary data of effects of cisplatin and after vitE pre-incubation obtained from *n* = 14 cells. **(D–F)** Outward K^+^ currents were elicited after K_v_1.4 transfection in HEK 293 cells. Cisplatin produced ∼45% reduction of the current density **(E)**. However, pre-incubation of vitE did not reverse the cisplatin-mediated reduction of the K_v_1.4-mediated outward current (**F**; *n* = 12 cells) (***p < 0.001).

K^+^ channel transcripts altered in TH^+^ DRGNs in vitamin E deficient mice are *Kcnd3, Kcna4, Kcnk18* (Finno et al., 2019), which encode for Kv4.3, Kv1.4, and K_2P_18.1 channels, respectively. We expressed human K_v_1.4 and K_2P_18.1 plasmids in the human embryonic kidney (HEK) 293 cell line to identify potential K^+^ channels responsible for altering the cisplatin-mediated effects. The cisplatin-sensitive current’s kinetic was non-inactivating and active at the resting membrane potential (Figures 5B, 6). We excluded the Kv4.3 channel from the list because the current shows inactivation kinetics, and it is primarily a cardiac ion channel (Dixon et al., 1996; Wu et al., 2010). HEK 293 cells transfected with K_2P_18.1, and Kv1.4 plasmids yielded outward currents compared to non-transfected cells. Transfected cells were assessed with and without cisplatin before and after pre-incubation with vitamin E. While cisplatin reduced the current density in K_2P_18.1 and K_v_1.4 transfected HEK 293 cells (Figure 7), pre-incubation with vitamin E only abolished the effects of cisplatin on the K_2P_18.1 current (K_2P_18.1; *p* < 0.001 and K_v_1.4; *p* = 0.94 Figures 7C,F, respectively). The neuroprotective effects of vitamin E on cisplatin-induced reduction of outward currents may be mediated through the K_2P_18.1 channel.

## Discussion

The etiology and targeted DRGNs underlying exaggerated mechanosensitivity following cisplatin chemotherapy remain a puzzle since targeted degeneration of only large-myelinated DRGNs may not account for the mechanism underlying CIPN (Starobova and Vetter, 2017; Zajaczkowska et al., 2019). Previous reports show that, within the mechanosensitive subset of TH^+^ DRGNs, two transcriptionally distinct classes emerged (Finno et al., 2019; Renthal et al., 2020). The heterogeneity of TH^+^ DRGNs was revealed further by the functional classification into three distinct neurons, namely fast (1-AP), moderately (>2-AP) adapting, and spontaneously active (SA) neuronal subtypes. The differential effects of cisplatin in increasing the membrane excitability of the fast and moderately adapting TH^+^ DRNGs, while the response properties of the SF neurons are impervious to the drug, raise the certainty that the TH^+^ DRGNs are not homogeneous. Current analyses show that vitamin E may curb cisplatin’s effects on mechanosensitive TH^+^ DRGN excitability. However, vitamin E may not reverse the loss of mechanical encoding properties of the neurons. A candidate K^+^ channel through which cisplatin may confer neuroexcitatory actions and the apparent protective effects of vitamin E is the two-pore K^+^ channel, K_2P_18.1.

Previous studies clustered the unmyelinated TH^+^ DRGNs into one population (Usoskin et al., 2015; Li et al., 2016). This earlier research identified the TH^+^ expression in the small neuronal C5-1 and C6-1 subpopulations (Li et al., 2016). These neurons were also *Mrgprd*^+^, which firmly defined the non-peptidergic subpopulation (Finno et al., 2019). However, a more recent study using single-nucleus RNA-sequencing also identified two C-LTMR populations (Renthal et al., 2020). The more definitive C-LTMR population was *Th^+^/Fam19a4^+^* (cLTMR1) population and a putative second population was *Fam19a4*^+^ but lower in *Th* expression and classified as p_cLTMR2. Of note, the p_cLTMR2 subpopulation had the most differentially expressed genes across all DRGN populations in mice administered the chemotherapeutic paclitaxel (Renthal et al., 2020). Based on expression profiles, the p_cLTMR2 group correlates to our TH2 population, supporting that differential responses exist to chemotherapeutics and vitamin E in these two C-LTMR subpopulations.

The appearance of two TH^+^ C-LTMR populations may not occur until later in postnatal development. Early studies that clustered the unmyelinated TH^+^ DRGNs into one population (Usoskin et al., 2015; Li et al., 2016) used mice from 6 to 10 weeks of age. When single-cell RNA-sequencing was performed across somatosensory neurons from embryonic day 11.5 to post-natal days 28–42 (i.e., 4–6 week old mice), only one C-LTMR population was identified (Sharma et al., 2020). This is in contrast to our study, which used 30-week old mice (Finno et al., 2019) and the recent 2020 study that used 8–12 week old mice (Renthal et al., 2020). While transcriptional subsetting may not be identifiable until later in postnatal development in mice, our current functional studies were performed on TH-EGFP+ transgenic were from 6 to 8 week-old mice. A limitation of our transgenic mouse model is that, in the initial studies that developed TH-EGFP+ transgenic mouse line, GFP expression was only evaluated during ventral midbrain development (Matsushita et al., 2002). Midbrain dopaminergic TH^+^ neurons demonstrated initial activation at early embryonic stages and reactivation during post-natal development under the 9-kb promotor of the rat TH gene that was used in this study (Matsushita et al., 2002). While clearly recapitulating endogenous TH expression in the ventral midbrain during development, this study did not evaluate DRGNs. Therefore, we first confirmed that GFP^+^ DRGNs were, in fact, TH^+^ C-LMTRs. Our subsequent findings that functional subsets of these TH^+^ C-LTMRs appear to exist at 6 weeks of age, with transcriptional distinction between subtypes evident after 10 weeks of age, requires further validation studies using protein-specific markers for TH1 and TH2 subtypes.

In the mouse, a distinct population of TH^+^ DRGNs, constituting 10–14% of DRGNs and located primarily in the lumbar DRG, innervate a portion of colorectal and bladder neurons (Brumovsky et al., 2012). This subset of neurons is positive for CGRP, unlike the non-visceral TH^+^ C-LTMR subpopulation (Brumovsky et al., 2012). While not included in the primary transcriptional TH1 and TH2 subsets, the SA neuronal subset may innervate the smooth muscles.

The three functional TH^+^ DRGN subtypes had distinct coding properties and are likely to have different roles in sensation. The fast-adapting 1-AP TH^+^ DRGNs had the lowest overall amplitude, input resistance, and the best temporal coding (i.e., high VS). These 1-AP DRGNs are time-coding neurons and would likely result in a fast-acting response to touch. Moderately-adapting ≥2-APs TH^+^ DRGNs fired ≥2-APs, with overall amplitudes, input resistance, and resting membrane potentials similar to the SF TH^+^ DRGNs, but lower current densities than both 1-AP and SA subtypes. Temporal coding of ≥2-APs increased at higher stimulation frequencies but remained lower than 1-AP TH+ DRGNs. These ≥2-APs neurons would likely result in a moderate response to touch. Lastly, the SA TH+ DRGNs would likely produce a slow reaction to the mechanical sensation as these are rate-coding neurons. While our current studies imply a strong correlation between TH2 and 1-AP TH^+^ DRGNs, targeted protein markers of TH1 and TH2 DRGNs are required, along with *in vivo* animal models with specifically deleted neuronal subpopulations, to definitively link the molecular subtypes (i.e., TH1 vs. TH2) to these functional subtypes (1-AP vs. ≥2-APs vs. SA).

The platinum derivate chemotherapeutic agents, cisplatin and oxaliplatin, produce painful peripheral neuropathies as dose-limiting side effects. Cisplatin damages all types of myelinated fibers (Boehmerle et al., 2014). In particular, large-diameter myelinated DRGNs (Cavaletti et al., 1992; Krarup-Hansen et al., 1999, 2007; McDonald et al., 2005) are more susceptible. A recent study using *Vglut3*^–/^*^–^* mice, which lack TH^+^ DRGNs and thus C-LTMRs, demonstrated that Vglut3 cells are necessary to express mechanical hypersensitivity in oxaliplatin-induced neuropathy (Draxler et al., 2014). We have shown that cisplatin differentially affected TH^+^ DRGNs, with no effect on SA subtypes but a reduction in outward current in both 1-AP and ≥2-APs TH^+^ DRGN subtypes. Cisplatin increased SF and significantly reduced VS, suggesting stimulus-response temporal coding in fast-adapting 1-AP TH^+^ DRGNs declines.

The putative mechanisms of the neurotoxicity associated with cisplatin chemotherapy include binding to mitochondrial DNA, leading to altered mitochondrial function and release of reactive oxygen species, changes in axon morphology, leading to sensory-motor axon degeneration, and/or chelation of extracellular calcium, resulting in altered calcium homeostasis (Starobova and Vetter, 2017). As a potent antioxidant, the neuroprotective effects of vitamin E against CIPN have been previously documented but were focused primarily on the effects on large-diameter myelinated DRGNs (Cavaletti et al., 1992; Leonetti et al., 2003; Pace et al., 2003). *In vitro* pre-incubation with vitamin E blocked the cisplatin-induced neuronal excitability in fast-acting 1-AP TH^+^ DRGNs, without restoring vector strength. With the discovery that vitamin E provides a protective effect against cisplatin-induced neurotoxicity within TH^+^ DRGNs, other agents that target these DRGN subtypes could be investigated to alleviate the neurotoxic effects of CIPN.

We provide evidence that the neuroprotective effects of vitamin E on cisplatin-induced CIPN may be modulated through K_2P_18.1 (Kcnk18). Knck18 encodes the TWIK-related K^+^ channel (TRESK), a K_2P_ channel with a prominent role in pain pathways. The pharmacologic inhibition of TRESK induces spontaneous pain behavior (Tulleuda et al., 2011). In humans, a dominant-negative frameshift mutation in *KCNK18* segregates in patients with migraines (Lafreniere et al., 2010), and a causal role for this loss of function mutation was recently established (Pettingill et al., 2019). TRESK heterodimerizes with two distantly related K_2P_ channels, TREK1 and TREK2 (Royal et al., 2019). Decreased expression of TREK1 was identified after treatment with a related platinum-based chemotherapeutic, oxaliplatin, in murine DRGNs (Descoeur et al., 2011). While not a defining subpopulation-defining transcript of the TH^+^ C-LTMRs (Finno et al., 2019; Zheng et al., 2019; Sharma et al., 2020), in our previous studies using a vitamin E deficiency mouse model, Kcnk18 was significantly upregulated in multiple DRGN subpopulations, including peptidergic and TH^+^ DRGNs (Finno et al., 2019). Thus, TRESK, and its interaction with TREK1, may be potential targets across DRGN subpopulations of cisplatin neurotoxicity. Functionally, we focused on KCNK18 as a potential target for cisplatin-induced effects. Still, since we did not perform additional experiments using heterologous expression systems to target other specific K2P channels, it is conceivable that other K2P channels are involved that this report has not evaluated and can be addressed in future studies. The neuroprotective role of vitamin E could be in maintaining the baseline excitability of this channel.

Despite early results that supported a protective role for vitamin E in the presentation of CIPN (Pace et al., 2003, 2010; Argyriou et al., 2005, 2006), a large-scale randomized clinical trial in 2011 refuted these results, concluding that vitamin E was ineffective in preventing CIPN (Kottschade et al., 2011). However, the 2011 clinical trial consisted of CIPN induced primarily by taxanes, whereas the earlier clinical trials specifically investigated the use of vitamin E in preventing cisplatin-induced CIPN. Additionally, the trials administered vitamin E at the onset of chemotherapy, rather than initiating supplementation before chemotherapy, similar to the design used in most animal studies (Leonetti et al., 2003) and analogous to pre-incubation with vitamin E in the *in vitro* experiments. A meta-analysis demonstrated that, while vitamin E supplementation did not decrease the overall incidence of CIPN, vitamin E significantly prevented cisplatin-associated neurotoxicity (Huang et al., 2016). Thus, while the current literature suggests vitamin E provides a minimal clinical improvement in CIPN (Hu L. Y. et al., 2019), early and targeted vitamin E supplementation to prevent cisplatin-associated peripheral neuropathy requires investigation.

We have identified three functionally distinct TH^+^ DRGNs with differential responses to cisplatin. The addition of vitamin E reduced the cisplatin-mediated increased excitability of 1-AP TH^+^ DRGNs but did not improve the temporal coding properties. The neuroprotective effects of vitamin E on CIPN may be modulated through K_2P_18.1 *(Kcnk18*) across DRGN subpopulations. The study provides a potential therapeutic target in patients receiving cisplatin chemotherapy.

## Data Availability Statement

The raw and processed data for scRNA-seq individual libraries have been deposited in the NCBI Gene Expression Omnibus (GEO) under ID codes (GEO:GSE128276, GSM3670444, GSM3670445, GSM3670446, GSM3670447, GSM3670448, and GSM3670449).

## Ethics Statement

Animals were housed and cared for under the University of California Davis (UCD) and University of Reno (UNR) standing committee on animal use and care (IACUC) as well as the Guide for the Care and Use of Laboratory Animals (8th edition, 2011). All procedures performed were also approved by the University (UCD and UNR) IACUC.

## Author Contributions

CF and EY were responsible for the conceptualization, funding acquisition, methodology, investigation, formal analysis, resources, and manuscript writing. YC, SP, JL, JC, and MP-F contributed to the study and research of the results. All authors have reviewed the final manuscript.

## Conflict of Interest

The authors declare that the research was conducted in the absence of any commercial or financial relationships that could be construed as a potential conflict of interest.

## Publisher’s Note

All claims expressed in this article are solely those of the authors and do not necessarily represent those of their affiliated organizations, or those of the publisher, the editors and the reviewers. Any product that may be evaluated in this article, or claim that may be made by its manufacturer, is not guaranteed or endorsed by the publisher.

## Footnotes

1
http://pantherdb.org/
